# PoPLAR: Portal for Petascale Lifescience Applications and Research

**DOI:** 10.1186/1471-2105-14-S9-S3

**Published:** 2013-06-28

**Authors:** Bhanu Rekapalli, Paul Giblock, Christopher Reardon

**Affiliations:** 1 Joint Institute for Computational Sciences, The University of Tennessee, Oak Ridge National Laboratory, 1 Bethel Valley Rd., Bldg. 5100, Oak Ridge, TN 37831-6173, USA

## Abstract

**Background:**

We are focusing specifically on fast data analysis and retrieval in bioinformatics that will have a direct impact on the quality of human health and the environment. The exponential growth of data generated in biology research, from small atoms to big ecosystems, necessitates an increasingly large computational component to perform analyses. Novel DNA sequencing technologies and complementary high-throughput approaches--such as proteomics, genomics, metabolomics, and meta-genomics--drive data-intensive bioinformatics. While individual research centers or universities could once provide for these applications, this is no longer the case. Today, only specialized national centers can deliver the level of computing resources required to meet the challenges posed by rapid data growth and the resulting computational demand. Consequently, we are developing massively parallel applications to analyze the growing flood of biological data and contribute to the rapid discovery of novel knowledge.

**Methods:**

The efforts of previous National Science Foundation (NSF) projects provided for the generation of parallel modules for widely used bioinformatics applications on the Kraken supercomputer. We have profiled and optimized the code of some of the scientific community's most widely used desktop and small-cluster-based applications, including BLAST from the National Center for Biotechnology Information (NCBI), HMMER, and MUSCLE; scaled them to tens of thousands of cores on high-performance computing (HPC) architectures; made them robust and portable to next-generation architectures; and incorporated these parallel applications in science gateways with a web-based portal.

**Results:**

This paper will discuss the various developmental stages, challenges, and solutions involved in taking bioinformatics applications from the desktop to petascale with a front-end portal for very-large-scale data analysis in the life sciences.

**Conclusions:**

This research will help to bridge the gap between the rate of data generation and the speed at which scientists can study this data. The ability to rapidly analyze data at such a large scale is having a significant, direct impact on science achieved by collaborators who are currently using these tools on supercomputers.

## Background

The data generated by various scientific and non-scientific fields is growing exponentially with modern technologies. This paper focuses on the exponential growth of data in the life sciences, which has surpassed the rate at which both processing power and storage technologies are growing [1]. In this work we focus on bioinformatics, a key area of information processing that has a direct impact on the quality of human life. Since the data in bioinformatics is growing beyond the scope of a single computing architecture, we are working on developing efficient, optimized, and highly scalable parallel applications on the latest and next-generation supercomputing architectures to meet the rising demand. The previously mentioned challenges relative to data growth and large-scale knowledge discovery could be addressed in three phases: the first phase is the development of parallel applications to analyze data at a rapid rate; the second is the creation of methods for easy access to these parallel applications; and the third is the development of databases to host analyzed data for quicker retrieval.

### Development of parallel applications

The software parallelizations that can be explored to address these gigantic problems are data parallelism, functional parallelism, and a combination of both data and functional parallelism. This paper discusses software wrappers used for data parallelization to process large-scale data in less time. These parallel wrappers aid us in parallelizing the most widely used applications in bioinformatics. The development of parallel applications is not the only aspect of the solution required for this data growth problem. After these applications have been developed, creating interfaces that will allow researchers with limited computing expertise to access these tools to solve their data analysis problems will also be essential. For this reason, we are constructing a science gateway with a web interface to expose these parallel applications for easy access. Finally, we are also developing a suite of tools to parse the outputs from all of the parallel applications and store the data in databases for faster retrieval and knowledge discovery since few of these tools generate more output data than input data by an order of magnitude or more.

Many parallel bioinformatics tools designed for large-scale data analysis exists. These implementations range from clusters to supercomputers and from grids to cloud computers. Wide ranges of workflows, gateways, and packages for various bioinformatics tools are also available, but implementations of highly scalable parallel bioinformatics applications with science gateways for large-scale data analysis are few to non-existent.

To build workflows needed by bioinformatics labs in the states of Tennessee and South Carolina as a part of an NSF proposal, we have identified the most widely used bioinformatics tools for sequence similarity searches, multiple sequence alignments, protein domain analysis, and phylogenetic tree construction. These tools include NCBI BLAST: Basic Local Alignment Search Tool [2]; HMMER [3] by HMMI Janelia Farm; MUSCLE: Multiple Sequence Comparison by Log-Exception [4]; ClustalW [5]; and MrBayes [6]. Many parallel implementations of these tools focused for clusters exist, such as mpiBLAST [7], ScalaBLAST [8], mpiHMMER [9], ClustalW-MPI [10], and Parallel MrBayes [11]. These tools achieve scalability by selecting techniques such as database fragmentation, parallel input and output (I/O), load balancing, and query prefetching, but only a few highly scalable implementations capable of using tens of thousands of cores on supercomputers are available, such as BLAST on Blue Gene/L implementation [12], pioBLAST [13], HSP-HMMER [14,15], and HSPp-BLAST [16]. By creating these highly scalable parallel tools and making them available to researchers through a science gateway, we are making a significant contribution to solving the pressing analysis needs in these areas.

### Science gateways

Many research groups have active science gateway portals as part of the state-of-the-art Extreme Science and Engineering Discovery Environment (XSEDE) NSF program. These gateways focus on fields such as biochemistry [17,18], biomedical computation [19], protein structure and interaction [20], and systemic and population biology [21]. These portals offer multiple tools for their respective areas and run a subset of those tools on machines that are best suited for HPC computational resources. For example, of the dozens of phylogenetic tools available on CIPRES, versions of MrBayes, RAxML, GARLI, and BEAST use XSEDE resources [21], and Robetta is designed to run on computer clusters distributed as mirrors [22].

Many portals have workflow capabilities for bioinformatics tools, such as Galaxy [23]. Some XSEDE informatics tools have available workflows, like ChemBioGrid [18], which has web-based computational workflows built on the Taverna [24] workflow tool. Currently, no portals exist that combine workflow between highly scalable bioinformatics tools on HPC resources with gateway access, along with tools for output data analysis. Our science gateway, the Portal for Petascale Lifescience Applications & Research (PoPLAR), will provide easy access to this unique combination of powerful, highly scalable parallel bioinformatics applications, output analysis tools, and knowledge-discovery resources. Our efforts to provide a new solution to this important problem are described in the section on development of parallel applications.

## Methods

In this section, we focus on describing the process to take a desktop bioinformatics application, scale it to tens of thousands of cores on supercomputers, and expose it to a science gateway for easy access. We look at the complexity of the code and various profiling options. We also identify the computationally intensive and data intensive sections of the code, and examine parallelizing the code with no change in the functionality of the application as described in following sections.

### Profiling for parallelization

The unique design of petascale supercomputers, with performance exceeding one petaflop, tens of thousands of computing cores, low per-core systems memory, and a reliance on networked distributed filesystems makes the architecture different from clusters and desktop machines. Efficient use of these machines requires special operating systems and carefully designed and tuned applications. One possible way to improve the performance of tools that run on petascale computers is by changing the actual algorithms to better suit the characteristics of supercomputers; this approach involves the tedious process of recoding already complex and specialized tools. The alternative approach that we have taken is to profile the code so as to identify the computationally intensive functions and I/O intensive functions.

We use CrayPat [25], PAPI [26], gprof [27], and our own timing code to analyze the runtime of the tools and eventually our own optimizations as well. The CrayPat profiler was useful in determining possible improvements to the data output scheme and improvements to the overall MPI communication. A second profiler, gprof, was used to analyze the runtime of the tools themselves. A great deal of source code is involved with each application; for example, NCBI's BLAST code base is approximately 1.3 million lines, HMMER at 35,000, and MUSCLE with nearly 28,000 lines. Because of the extensive code base of these tools, as few changes as possible should be made to the code so as to avoid the need to maintain millions of lines of forked software.

**Figure 1:**
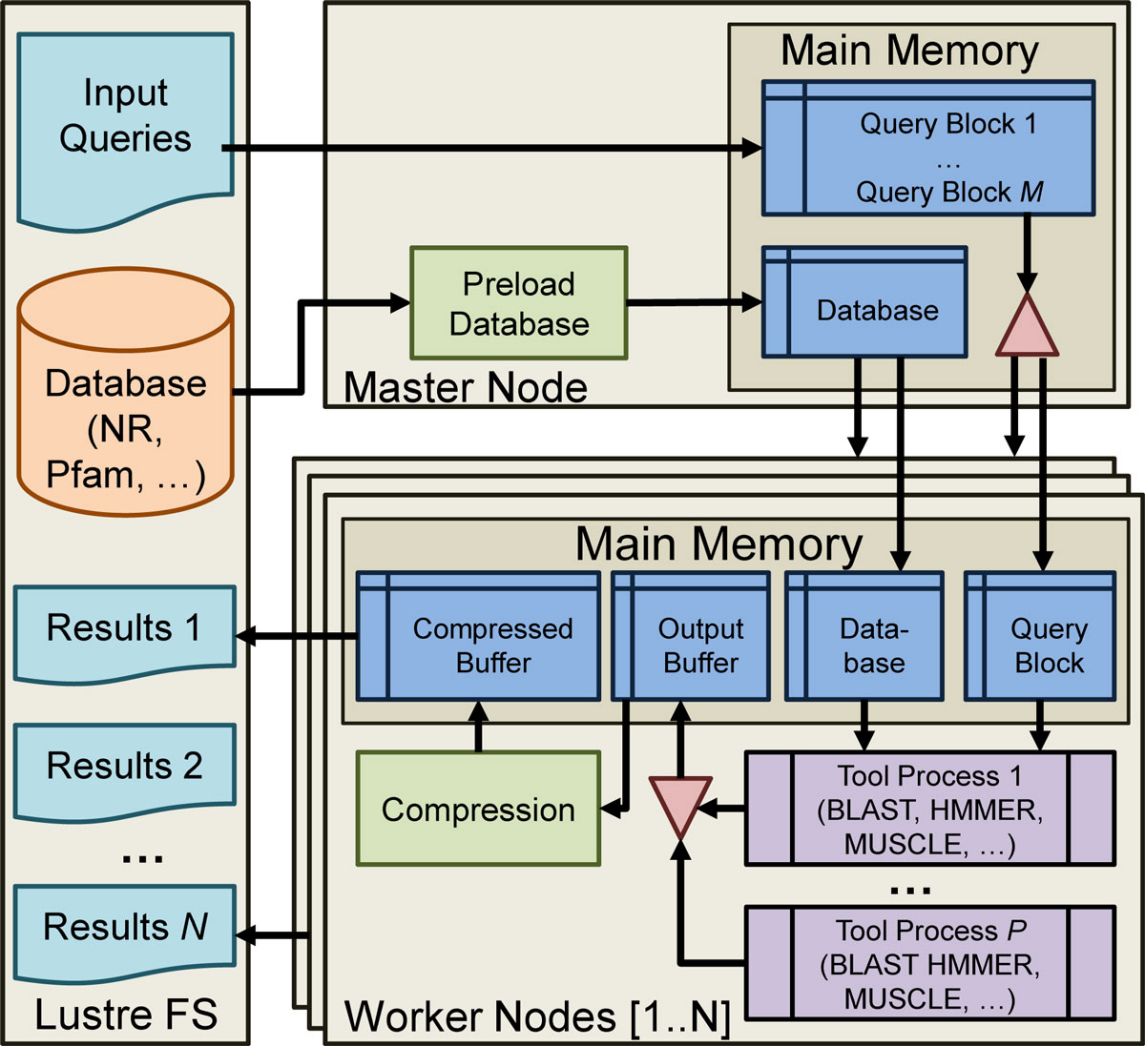
**HSP wrapper architecture**. The parallel wrapper used to scale widely used bioinformatics tools on HPC architectures.

After profiling and examining thousands of lines of code, we decided not to alter the functional part of any of these tools so we could avoid the time investment necessary for understanding and parallelizing the tool for advanced architectures. Instead, we created a software wrapper that requires only a very small number of changes to each tool's original source code. The architecture of the wrapper solution is shown in Figure 1. The wrapper is an executable that runs outside of BLAST, HMMER, and MUSCLE, but serves to handle the I/O of these tools in an efficient manner, leaving the core of the application as an untouched "black box". The main difficulty in scaling these tools is management of I/O access patterns. The wrapper uses shared memory to redirect the tool's I/O to the wrapper process, so that it may handle the I/O more efficiently. We chose shared memory segments as the means of inter-process communication due to its low overhead, the ease with which pseudo file I/O can be implemented, and the optimization opportunities it provides as described later. Many supercomputers use a distributed filesystem, such as the Lustre distributed filesystem [28], for I/O data storage. Each instance of the tool needs to load a copy of the database, but the file system does not perform well if thousands of processes are trying to read the same files from it simultaneously. A better design is to load all of the data needed with one reader and then broadcast the data to all of the worker nodes. This ensures that the data is only read from disk once. In our solution, we load data from the file system to the master node, and use MPI's MPI_Bcast function to broadcast data from the master node to all worker nodes that require the input databases and configuration files. This broadcast is more efficient, scaling logarithmically in the number of nodes. Additionally, the use of shared memory allows for a single image of the database to be shared by multiple processing cores. The worker nodes prefetch query sequences from the master node in order to hide input latency while achieving dynamic load balancing, which is essential due to the variance in query runtimes [16].

### Optimization for improved data access

Another common issue related to file access patterns is the reliance on sequential loading of files. In this model, a file is memory-mapped into the process's virtual address space and then read sequentially. A page fault occurs each time the program attempts to read data that has not yet been loaded from the filesystem. Then this next page of data is loaded into memory. This results in many small requests for data from the networked file system, which is slower than loading larger blocks of data. Our solution to this problem is to preload the database into memory with a single system call. The database typically consumes the most memory during a run. The large size of the database could be larger than the area addressable by the system's translation look-aside buffer (TLB) when standard-size pages are used. Therefore, we increase the page-size of this preloaded region so that it requires fewer entries in the system's TLB, which decreases the number of TLB cache misses. We found that the use of 2 MB page sizes instead of the standard 4 KB can reduce the processing time of the BLAST tool by 15% to 60% depending on the parameters used [16].

The performance of output operations was also improved in several ways. Originally, data would be written to disk immediately when available, resulting in many individual writes to the file system and plaguing performance. Our improvement consists of a two-stage buffering technique. In this scheme, the tools write output to an in-memory buffer instead of directly to disk. The data is flushed from in-memory buffers to disk by a background process when the buffers are nearly full, rather than on demand. This increases the output bandwidth and also results in more uniform output time. Writing to disk in the background also reduces blocking in the tool itself. Additionally, we distribute the output files across multiple directories to optimize the common scenario of architectures with a distributed file system. This optimization works because it increases the likelihood that the file system will locate the data on multiple hardware devices, resulting in higher bandwidth for parallel I/O. In addition to writing data in blocks, we provide the option of compressing the actual output blocks. Allowing output to be transferred and stored in compressed form helps to increase the throughput of output records. Compression occurs in the background so that no additional latency is introduced when the tool writes data. These output enhancements have shown an increase in output bandwidth of 2809% [16]. All the above optimizations are performed by a single wrapper [16], shown in Figure 1.

### Output analysis tools

**Figure 2:**
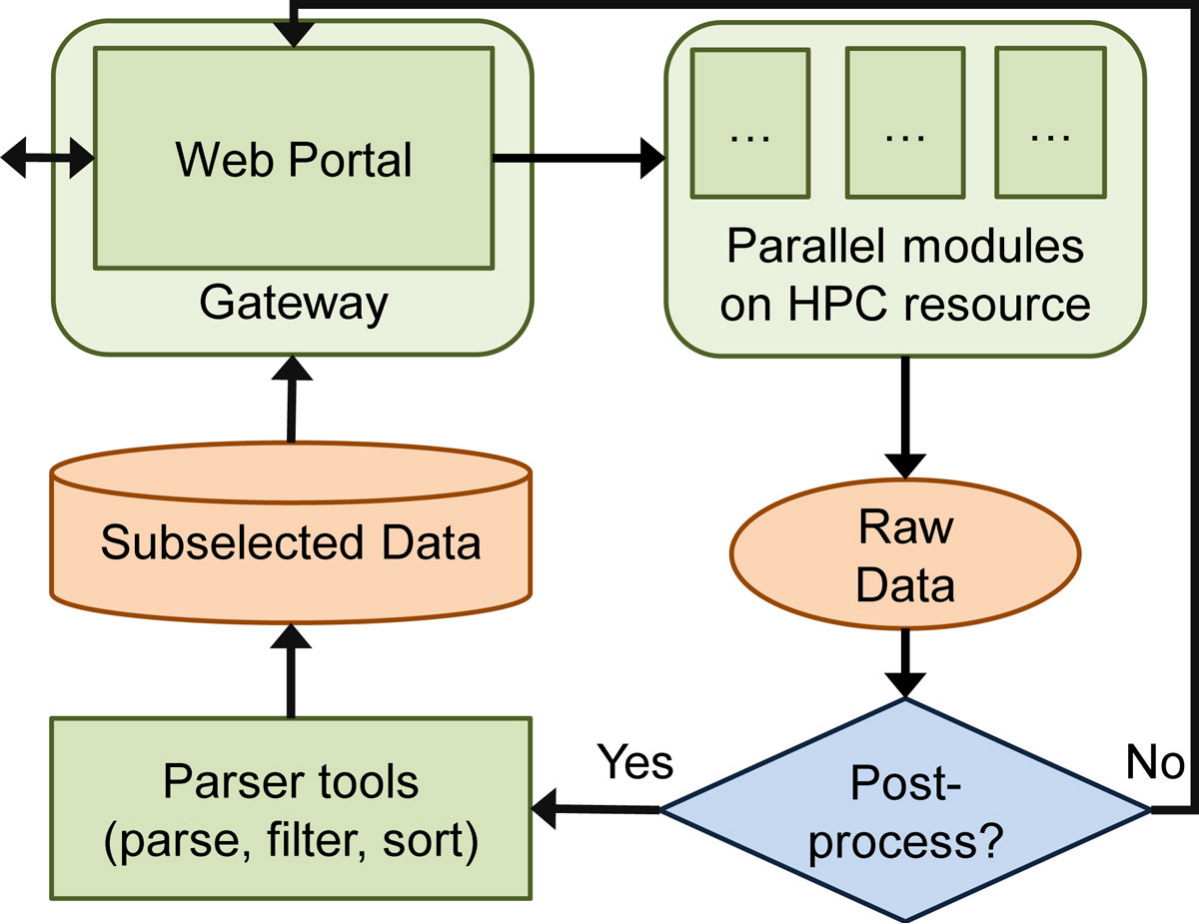
**The gateway approach**. The user interfaces with the science gateway portal and initiates a job that is run through parallel modules on an HPC resource. Based on the user's needs, either the raw results are delivered back to the user, or the output is parsed and prepared for easier use before it is made accessible through the gateway.

We have also developed large-scale output data analysis tools, because the data generated by these massive runs of query sequences could vary from gigabytes to terabytes and sometimes accessing and analyzing such large datasets is prohibitive. We have generated parsers to parse the XML [29] and other output formats into tab-delimited and user friendly outputs, along with generating SQL databases for easy retrieval of results. The user will have access to his or her data either in raw data formats that the tools generated or the parsed data formats if desired. All the results will then be made accessible through science gateways. Figure 2 illustrates this approach, where the user interfaces with the science gateway portal and initiates a job that is run through parallel modules on an HPC resource. Based on the user's needs, either the raw results are delivered back to the user, or the output is parsed and prepared for easier use before it is made accessible through the gateway.

### Science gateway: challenges and solutions

The usefulness of science gateways, which provide the ability to submit jobs to HPC resources remotely via a defined service, is well established [22]. By allowing scientists, researchers, and students to use HPC resources in this way, we can provide a user interface that is highly customized to the user's purpose. This saves time and effort on the researcher's part, and by regulating and standardizing the input interface, we use computing resources more efficiently through the streamlining of complex workflows and the reduction of obstacles caused by deficiencies of expertise and experience with computational resources.

In the process of creating the PoPLAR science gateway, we were faced with several design decisions that directly affected the performance and functionality of our end product. To provide a single experience accessible to the broadest audience, we have implemented PoPLAR as a web-based portal, rather than an alternative solution such as a thick client or desktop application. This also allows us to better centralize management and data flow, and to enforce requirements and best practices such as those for XSEDE resources.

The initial challenge when establishing a portal is the selection of an architecture that can fulfill the requirements of a science gateway and the needs of the project and its users. The different components have mostly out-of-the-box solutions. While many possible options exist, we identified three that most closely met our needs: the HUBzero [30], Galaxy [29], and CIPRES [27] platforms. HUBzero and Galaxy were the first two platforms we examined. They were both attractive solutions for a front-end, because they are feature rich, with built-in capabilities that fulfill many of the XSEDE gateway best practices (e.g., user registry with permissions, logging of users and usage, job monitoring, statistics tracking, system logging), and have powerful administrative interfaces. HUBzero is a general science collaboration environment that is very focused on allowing users to contribute tools, whereas Galaxy is more focused on biological sciences and has strong workflow construction functionality. Both have some version of submitting jobs to remote computational resources. From our perspective, the strength of HUBzero and Galaxy was also their weakness in that extra development time would be necessary to make them usable for our purposes; and generalizability could detract from our more focused research. The time investment was our primary concern for both relative to adapting and supporting the packages.

The third solution we examined and chose is an adaptation of the CIPRES Science Gateway (CSG) platform [31]. The CSG platform combines powerful, built-in capabilities and a focus on computational biology applications in such a way that it meets most of our feature requirements without over-scoping and requiring extensive customization. Some of the most attractive aspects of CSG included (a) a focus on HPC applications, specifically adapted to both XSEDE and academic resources; (b) a scalable architecture designed for fully exposing multiple applications (such as our Highly Scalable Parallel [HSP] tool suites) to users via an easy-to-use graphical interface; and (c) total customizability and parameterization of these tools.

CSG uses the Workbench Framework, which is a "generic distributable platform to access and search remote databases and deploy jobs on remote computational resources" [31]. The Workbench Framework implementation provides a scalable mechanism of XML descriptions that map to graphical user interfaces (GUIs); and furnishes a schema for constructing command line statements with the user input entered into those GUIs [21]. This approach offers scalability via ease of development, a robust mechanism for specifying, capturing, and error-checking user parameters, and abstracting presentation from content to allow for separate manipulation and development of both.

For those reasons and because CSG so closely matches our application needs, we selected it and thus were able to reduce initial implementation time and focus on customizing and extending the framework.

One of the biggest challenges we aim to address is the transfer of data. Our system allows for multiple runs of jobs--in parallel--on very large bioinformatics data sets. When using these tools locally on an HPC resource, the resulting output is stored to the file system. The design of this project requires the delivery of that output to the end user who has submitted the job via a web interface that is not physically co-located with the computation machine's file system. Therefore, scaling presents major challenges relative to handling large transfers and meeting local storage requirements. The synchronization of other job information with the user, such as job status, completion, errors, and so forth, also presents an issue. We have implemented a system and run jobs of moderate size via PoPLAR, and are continuing our efforts to improve the scalability of the system.

### Science gateway: architecture overview

**Figure 3:**
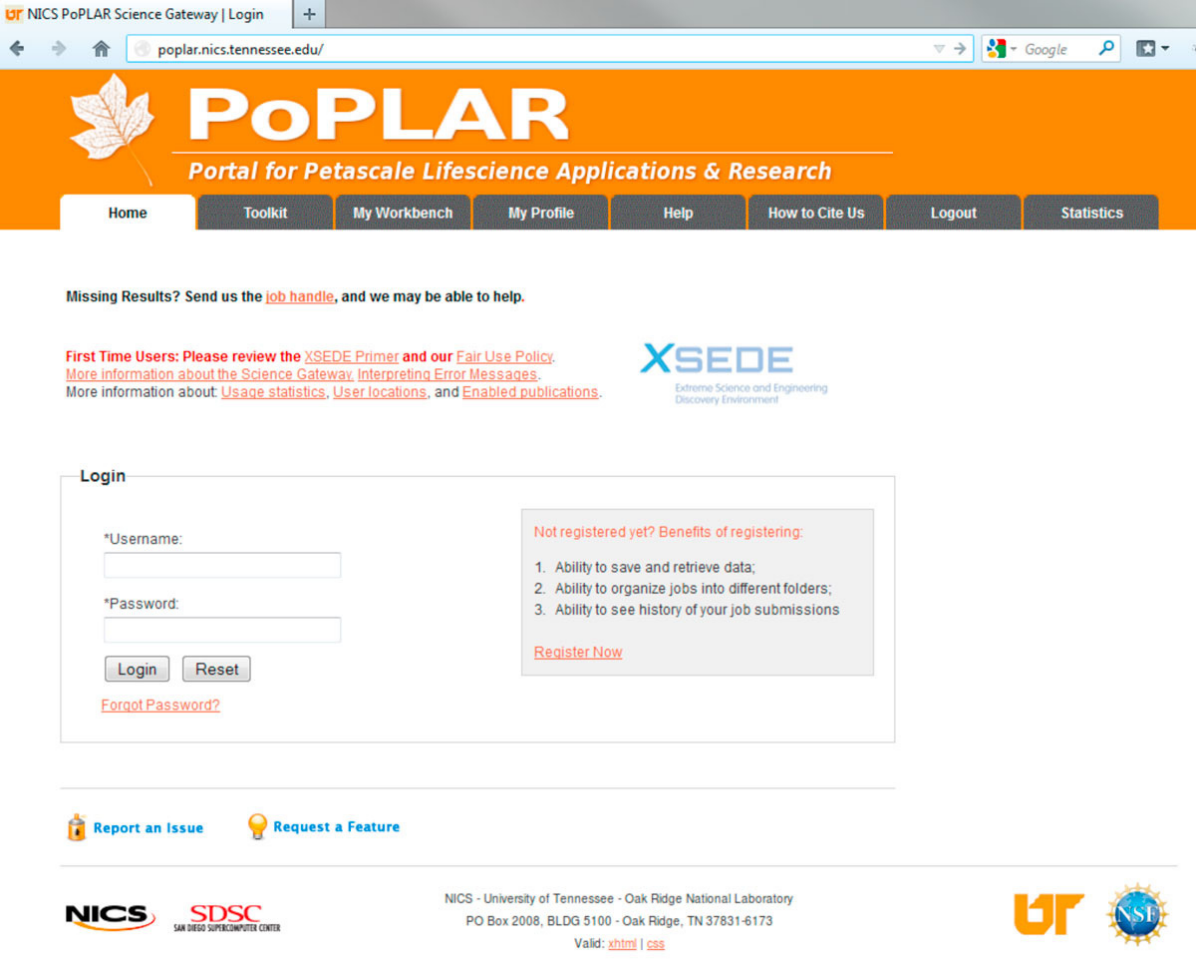
**PoPLAR science gateway login**. Example screenshot of the login interface.

**Figure 4:**
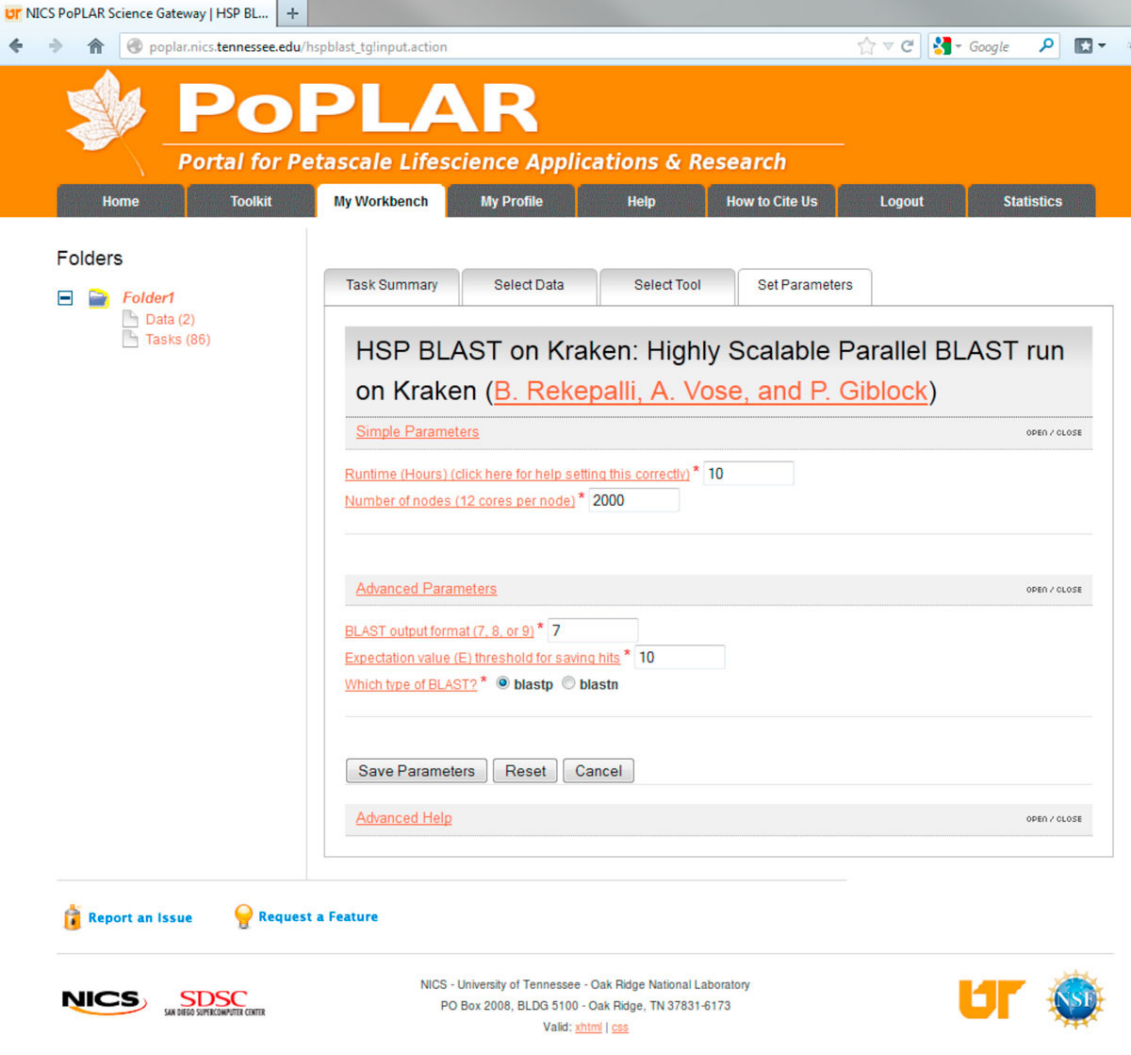
**PoPLAR science gateway tool parameters**. Example screenshot of the user configurable parameter setup options for the HSP-BLAST tool. In this figure, the user can specify runtime, number of nodes, and tool-specific parameters such as output format, threshold expectation value, and BLAST type (protein or nucleotide).

**Figure 5:**
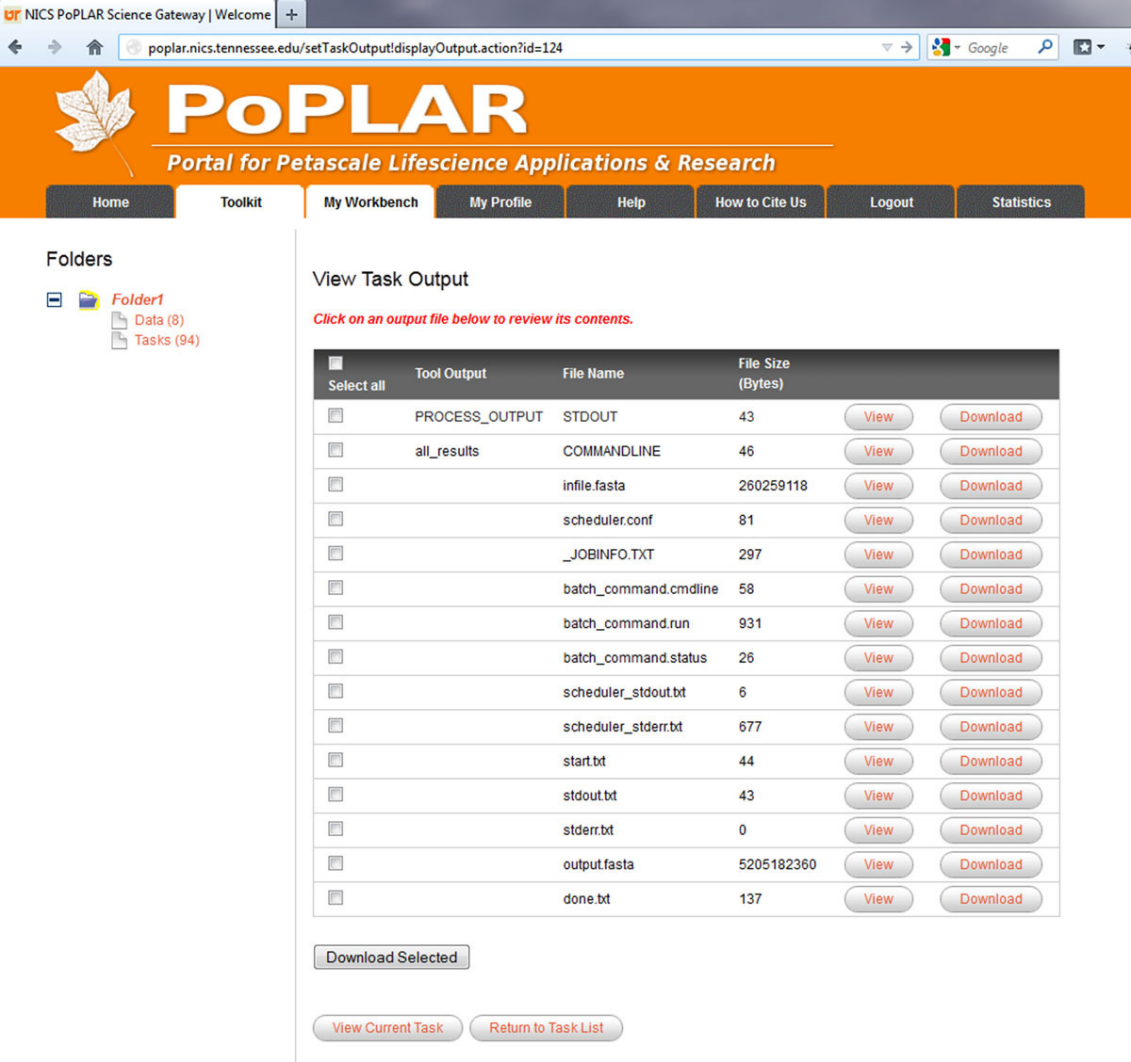
**PoPLAR science gateway output**. Example screenshot of a job output showing files available for the user to download. Here, the user can download results files (output.fasta) as well as other intermediate files generated during the job.

PoPLAR, our science gateway, uses the CSG framework [31] and incorporates a Java Struts2 [32] based web portal running on Linux and Apache Tomcat with a MySQL database and employs Python job management scripts on remote computational resources. Our implementation supports only registered users and restricts job submission to only verified users with activated accounts. The web interface allows users to upload data sets; and create, configure, and submit jobs using specific tools, with their uploaded input data and tunable per-tool parameter settings. After job submission, the system populates the results into the portal and notifies the user of job completion. Screenshots showing the login interface, an example user-configurable parameter setup for the HSP-BLAST tool, and output after a successful job, are shown in Figures 3, 4 and 5.

The system uses a community account for job submission and allows for individual user registration, authorization and authentication, detailed logging of portal and computation usage, submission of user attributes to compute resources, the ability to restrict or deny access to individual users, as well as system logging and other features. Our adaptations after implementation include restriction of access to registered users, the addition of a verification process for account activation, the enforcement of country of citizenship restrictions on access to computational resources, the adaptation of remote resource job maintenance scripts to the National Institute for Computational Sciences supercomputer, and changes in branding. We have incorporated several of our highly parallel scalable tools into the toolkit. We are working to extend the framework by incorporating integrated workflows across multiple tools, adding parsing and analysis tools as discussed above, and scaling data handling to hundreds of gigabytes for I/O.

## Results and discussion

### Scaling of parallel applications

**Figure 6:**
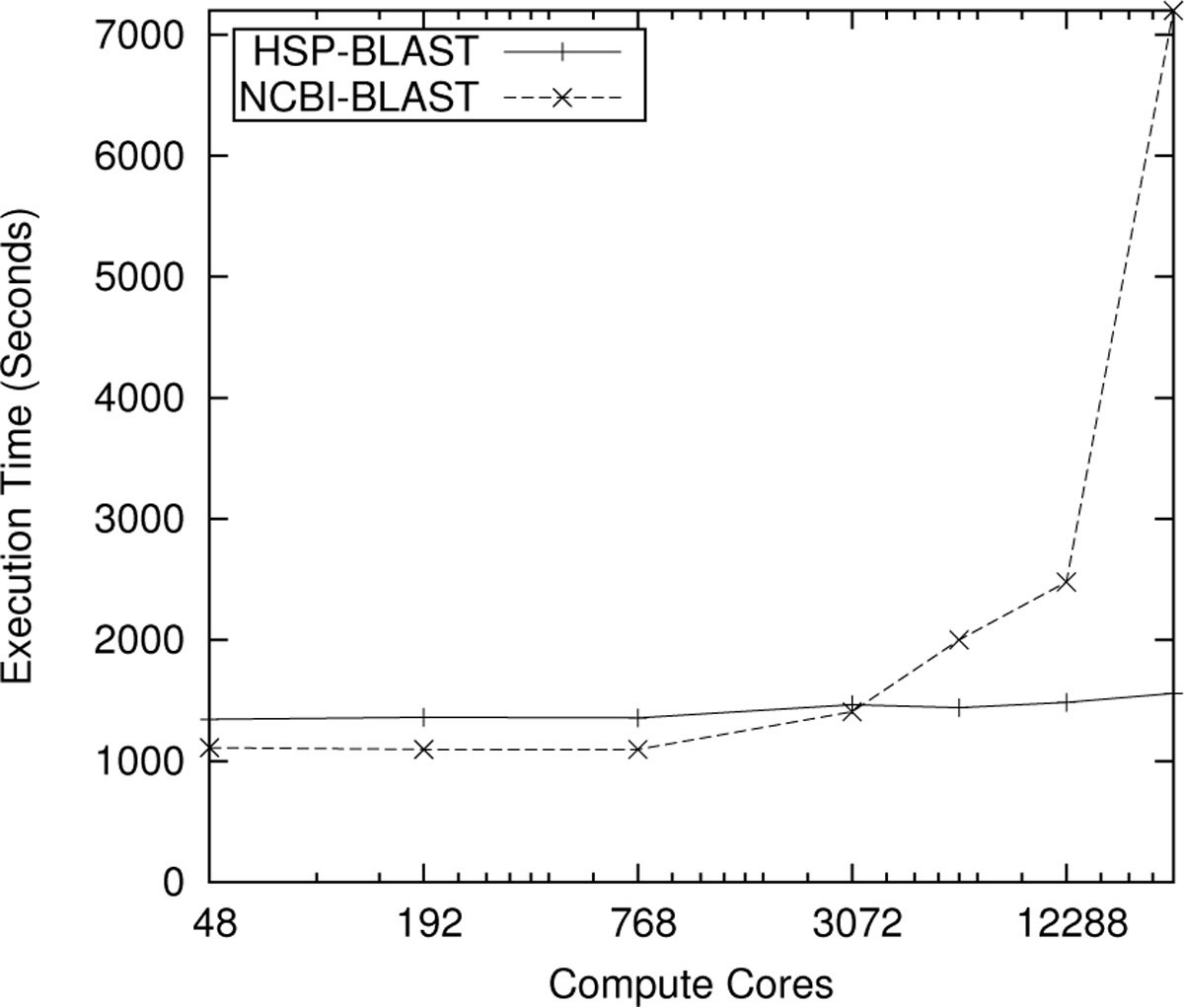
**Scaling comparison of HSP-BLAST and NCBI BLAST on Kraken**. Weak scaling results for BLAST Protein sequence similarity searches are performed up to 2048 nodes (24,576 cores) on the Kraken supercomputer.

Kraken supercomputer, an NSF-funded Cray XT5 machine consisting of 9,408 nodes with 112,896 AMD Opteron compute cores operating at 2.6 GHz with 147 TB of memory, was used for testing our HSP tools. Figure 6 shows the weak scaling results of running our HSP versions of BLAST (blastp) as compared to the unwrapped NCBI BLAST. The input database for BLASTP runs was NCBI's non-redundant (nr) protein database of March 2012. This database contains 17,577,257 protein sequences, and the total size of the formatted database is 11 GB. The query sequences are all 200 amino acids in length, and the number of sequences chosen is equal to 16 times the number of cores. The XML output format was used, and all other parameters were set to the default values established by the blastall tool. We tested the unwrapped BLAST by dividing the set of input sequences into a unique file for each compute core. Output is written to an individual file per core, arranged in a directory. Figure 6 shows the execution time (wall time) of HSP-BLAST compared to NCBI BLAST as the workload is increased in proportion to the number of compute cores. HSP-BLAST achieves near linear scaling performance while the NCBI version experiences scalability issues near 3000 cores. The original NCBI implementation does outperform HSP-BLAST at low core counts due to the lower startup cost. We also ran scaling studies of our wrapped Position Specific Iterative (PSI)-BLAST [16], whose performance was near linear. This is a significant accomplishment, as no parallel PSI-BLAST is currently available that scales to a large number of cores. We have run millions of BLAST searches in hours using ~240,000 cores on the Kraken supercomputer that would take weeks on a cluster or in a cloud environment.

**Figure 7:**
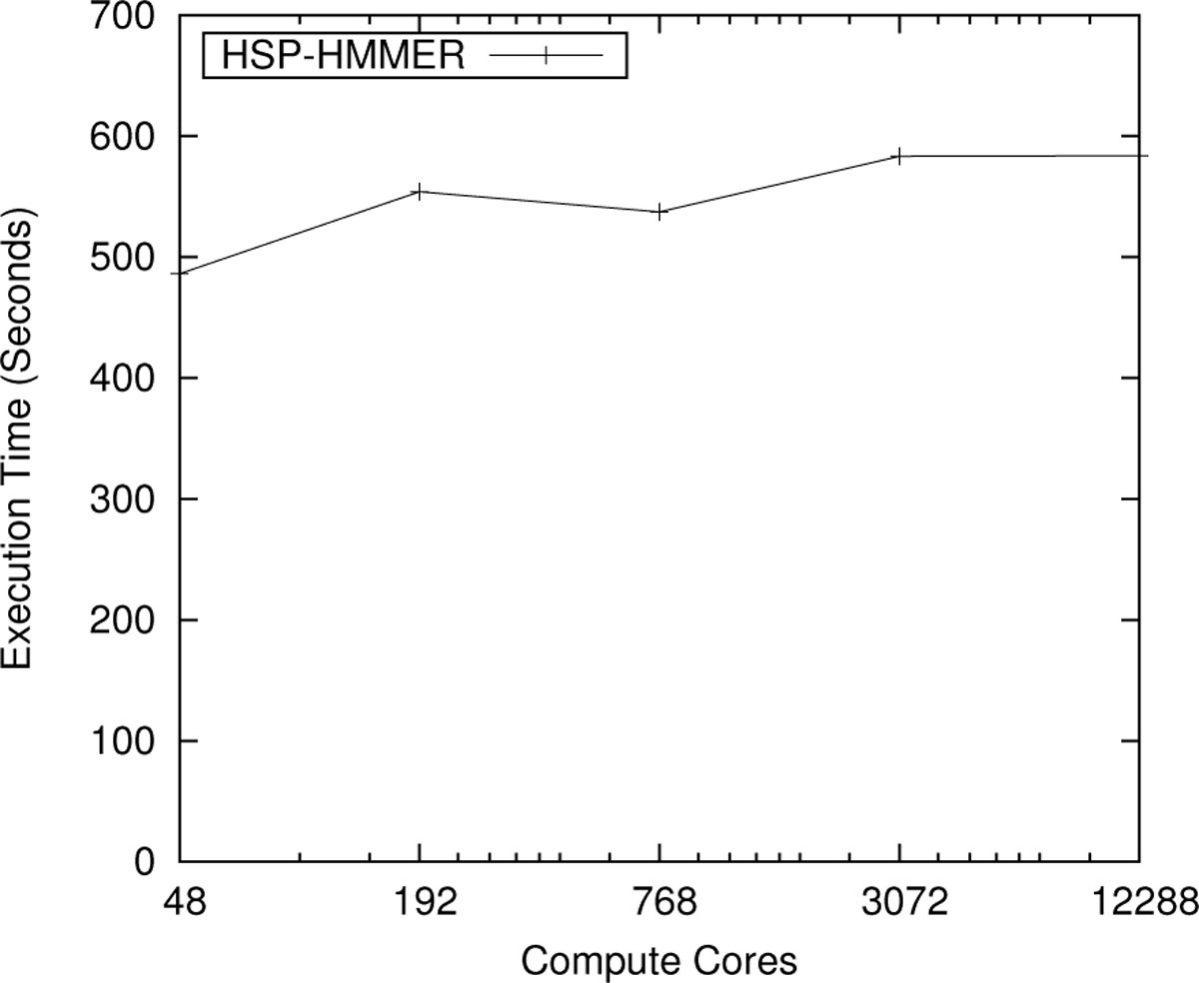
**Scaling study of HMMER on Kraken**. Weak scaling results for protein domain identification using the hmmscan function of HMMER are performed up to 1,024 nodes (12,288 cores) on the Kraken supercomputer.

Figure 7 shows the scaling results of the wrapped hmmscan function of the HMMER 3.0 package. The Pfam 24 database was used, and input sequences were chosen so that each computation core would align approximately 760 sequences each. The simple tabular output format was used. There is noticeable fluctuation in the execution time; particularly at 768 cores. This is due to the unpredictable performance of the shared Lustre file system. The load generated by other users of Kraken cause contention for the file system and directly affects the performance. Another scaling study that was published in the TG'11 conference scaled the wrapped HMMER tool to ~100,000 cores on the Kraken supercomputer [15], with near linear speedups; the tool is scalable up to a full capability mode run on parallel supercomputers such as Kraken. With HMMER, we were able to identify domain models for 10 million protein sequences in 10 minutes by using 100,000 cores on Kraken.

**Figure 8:**
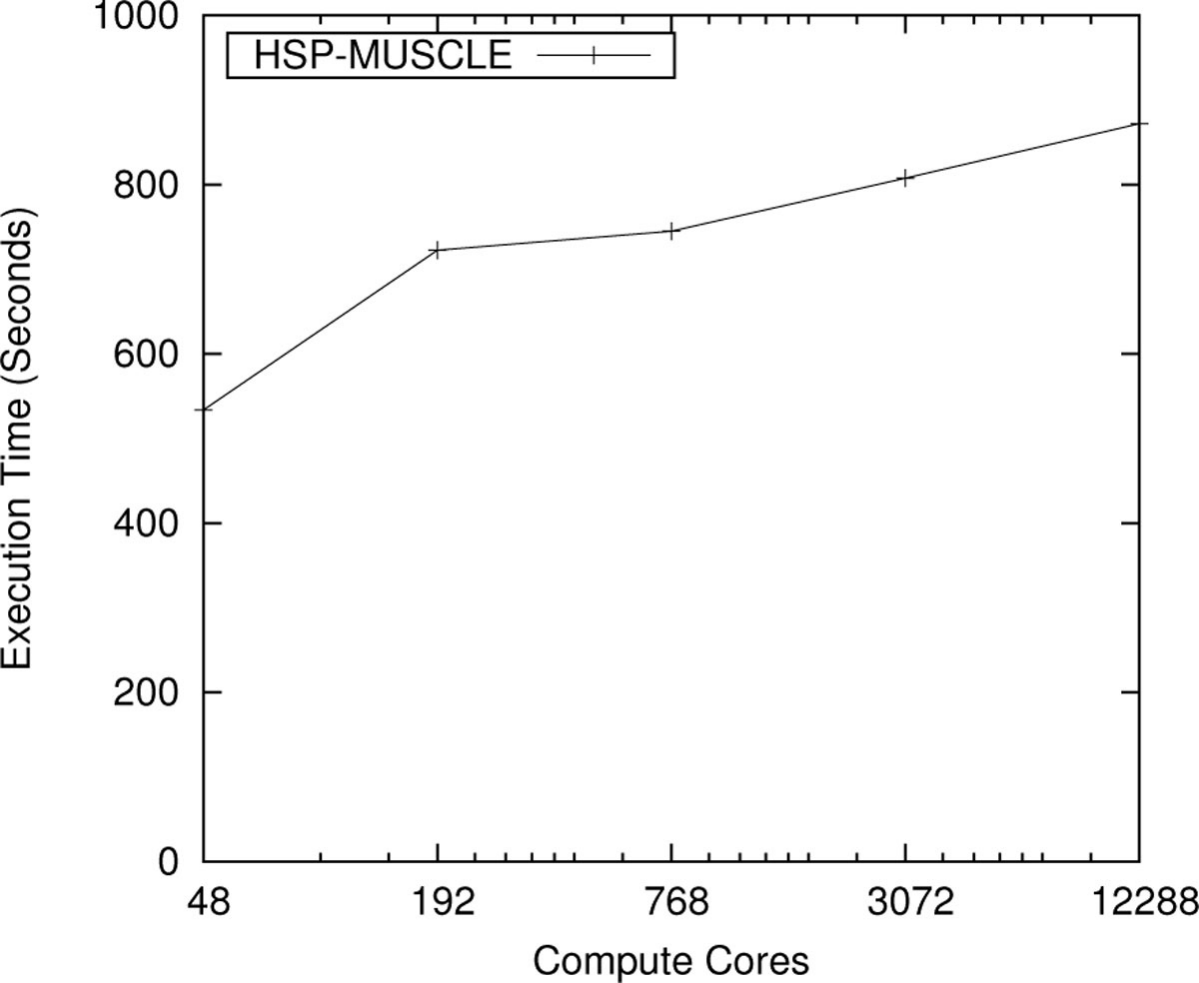
**Scaling study of MUSCLE on Kraken**. Weak scaling results for MUSCLE multiple sequence alignments are performed up to 1,024 nodes (12,288 cores) on the Kraken supercomputer.

The performance of the wrapped version of the MUSCLE multiple sequence alignment tool was also evaluated on Kraken. We chose 10 input data sets for each core used in the experiment. Each data set consisted of a number of sequences returned previously by a BLAST alignment search. Figure 8 shows the scaling results up to 12,288 cores or 1024 nodes on the Kraken supercomputer. We foresee only few users going beyond the core count. We scaled NCBI BLAST and HMMER to full machine runs on the Kraken supercomputer, and we scaled MUSCLE to 40,000 cores. These tools are accessible through our PoPLAR gateway.

### The PoPLAR gateway

**Figure 9:**
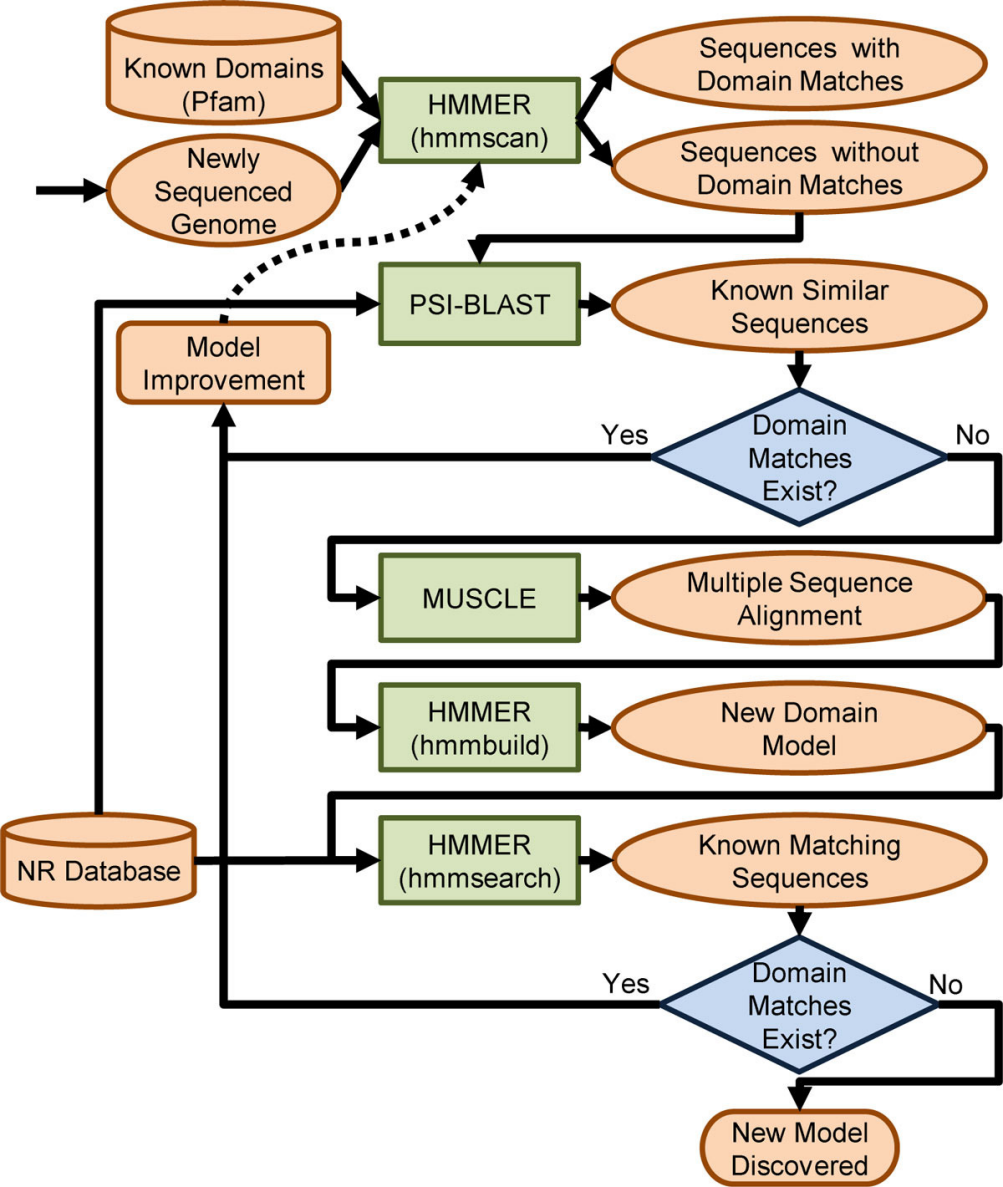
**An automated workflow for the discovery of novel domain models**. An example workflow created by combining bioinformatics tools to generate novel protein domain models on a massive scale.

We have generated modules for our wrapped tools on the Kraken supercomputer along with documentation for submitting jobs through command line. As discussed in the Implementation section, we have also developed our science gateway called PoPLAR. We have incorporated our parallel tools into the PoPLAR gateway and plan to add other bioinformatics tools during the development process. We present these tools via a science gateway so that researchers can use web portals to access supercomputers and further develop workflows of parallel tools that could analyze very large-scale life sciences data, without the need to learn command-line scripting. For example, we are developing a systems biology workflow for a bioinformatics lab at the University of Tennessee, Knoxville (UT-Knoxville), as shown in Figure 9. This workflow results from combining HSP bioinformatics tools currently available on PoPLAR to generate novel protein domain models at a massive scale. This workflow illustrates a real-world application that is commonly used by biologists at UT-Knoxville, as well as elsewhere, but must be run in a disjointed fashion, one tool at a time, which is difficult and demanding to perform on large data sets. Examples of currently implemented tools include BLAST, HMMER, and MUSCLE.

In this scenario, the researcher has a newly generated set of genomes or sequences to be annotated for domain models that determine the function, structure, and evolution of the proteins. First, the HMMER tool hmmscan is used to identify domain models in those sequences and those without domain matches. Ones with domain matches are identified and documented. To find sequences similar to those without domain matches, the next step is to use the NCBI PSI-BLAST tool. If similar sequences are found using PSI-BLAST to contain known domains, the models for those domain matches can be updated; thus, the next time HMMER should be able to identify the proper domain matches. If PSI-BLAST does not find sequences with domain matches, the next step is to build novel domain models. Before building a model, the matching domains are aligned with using tool like MUSCLE, which generates a multiple sequence alignment (MSA). The MSA is then used to build a new domain model with hmmbuild, part of the HMMER package. That domain model is checked against the known nr database using the HMMER tool hmmsearch. If hmmsearch generates domain matches, the existing models can be improved. If not, the researcher has confirmed that the model created is new and can now be added to domain databases around the world. By making a system where the I/O of each tool are reconfigurable, all the bioinformatics tools, result-parsing tools, and data-conversion tools of the workflow could be used in various combinations to solve different problems in biology.

The data transfer in our current system takes place between the end user and the gateway and between the gateway and the compute resource. A typical sequence begins with the user uploading an input set to the gateway. We note here that once uploaded, an input set is saved indefinitely for reuse, so the sequence may also begin with using a previously uploaded data set. Then, after the user selects a tool and configures its parameters, the gateway copies the input data to the compute resource using Globus tools. After the job completes, the gateway copies the results back, and the end user can view or download the data.

To examine the scalability of data transfer, we conducted a series of tests to determine the current limits of our system. We tested a variety of I/O file sizes, from 100MB to 5GB. Our results showed that for input, we are currently limited by our data upload capability; the maximum input size accommodated by our system is approximately 500MB. For output, we tested output sizes incrementally up to 5GB, without finding a size limitation. In the future we plan to address the upload limitation through software and hardware changes. We are also investigating methods to improve data transfer speeds and to eliminate the need to copy output back to the science gateway for user access. Finally, we are also adapting the gateway to allow users to easily apply output data as input into another tool.

The PoPLAR gateway provides an easy-to-use graphical interface that is more efficient than using the command line, for example, in several ways. It is worth noting that because the tools being run via PoPLAR and via the command line are identical, there is absolutely no difference from a processing perspective in the efficiency between methods of the tools themselves. Rather, the benefits PoPLAR provides are through the ease-of-use of the graphical interface as well as several functional advantages, including:

• The GUI replaces the command line interface, which frees the user from needing experience with the command line.

• Likewise, knowledge of each specific tool's syntax is not necessary, as each tool's parameters are broken out and presented via a web form with help text.

• The portal presents the user with one location for data (inputs and results), easy organization of data into folders, and a history of all jobs submitted.

• Users can submit and move on to other tasks, as notification of a job's completion is sent to the user via email.

• Different tools can be configured to run transparently on different computation resources, all made available to the user via the same interface.

• The computation-resource-agnostic interface does not require the user to know anything about the specifics of different systems (e.g., job submission engines, distributed filesystems, or command shells); the same tool running on different resources can be configured to present the same interface.

• Jobs submitted via the portal do not require the user to have an account on and log in to each computation resource (although the portal does allow users to charge their existing allocation)--only a browser is required to access the portal from anywhere.

By addressing the challenges described above and developing a web-portal science gateway for highly parallelized tools for large-scale data analysis, we look forward to having a substantial positive effect on these fields. Our approach allows for easy, user-friendly access to supercomputing resources. With that access, users can more easily submit large-scale jobs. By facilitating rapid large-scale analysis, we are able to help fulfill the significant demand created by the growing volume of data analysis needs in bioinformatics.

The system we have implemented is easily extended to incorporate similar highly scalable parallel tools for other domain sciences. By design, tools located on any computational resource can be made available easily and seamlessly through the science gateway. This combination makes our tools-plus-gateway approach powerfully scalable across both computational resources and science application domains.

## Conclusions

This paper provides a model for the development of highly scalable parallel bioinformatics applications on HPC architectures along with an increase in availability and usability through science gateways. The model directly enhances large-scale data analysis and knowledge discovery capabilities. This work revealed the following points: (a) Significant time and skills are required to change the entire code of an application to a specific architecture, and avoiding such an endeavor is the best practice; (b) a superior approach is to wrap the code of the bioinformatics application without changing functionality, identify the intensive I/O part of the code, and optimize communications to scale the code well, thus generating accurate results; (c) even though parallel tools are available at supercomputing facilities, many researchers are reluctant to use them due to a lack of expertise operating on the command line; (d) for these reasons mentioned here, developing science gateways with easy access to the parallel tools is a better way to facilitate and encourage the use of supercomputing resources by biologists. This research will have a direct impact on life sciences data and the rate of knowledge discovery. Still, many issues exist--such as job scheduling, load balancing, and fault tolerance--that need to be addressed with science gateways. We want our users to have total control over the type and size of data that can be analyzed, and we are addressing those issues in the life sciences gateway, as well as developing automated workflows for large-scale data analysis.

## Availability and requirements

**Project name: **PoPLAR

**Project home page: **http://poplar.nics.tennessee.edu/

**Operating system(s): **Web based / Platform independent

**Programming language: **Java

**Other requirements: **No

**License: **Contact author

**Any restrictions to use by non-academics: **None

## List of abbreviations used

CSG: CIPRES Science Gateway

GUI: graphical user interface

HPC: high-performance computing

HSP: Highly Scalable Parallel (tool suites)

I/O: input and output

MPI: Message Passing Interface

MSA: multiple sequence alignment

NCBI: National Center for Biotechnology Information

nr: non-redundant

NSF: National Science Foundation

PoPLAR: Portal for Petascale Lifescience Applications and Research

PSI: Position Specific Iterative

SQL: Structured Query Language

TLB: translation look-aside buffer

UT-Knoxville: University of Tennessee, Knoxville

XML: Extensible Markup Language

XSEDE: Extreme Science and Engineering Discovery Environment

## Competing interests

The authors declare that they have no competing interests.

## Authors' contributions

BR conceived and served as principal investigator for the project, created the layout of the project, analysis tools, gateways and authored sections of the manuscript. PG developed the highly scalable parallel tools and authored sections of the manuscript. CR implemented the PoPLAR science gateway, developed workflow tools, authored sections of the manuscript. All authors read and approved the final manuscript.

## Authors' information

BR is the research scientist at the Joint Institute for Computational Sciences (UT-Knoxville-Oak Ridge National Laboratory) who is developing parallel bioinformatics applications on HPC machines and next-generation architectures, along with collaborating with researchers from various universities on large-scale data analysis in life sciences. PG and CR are graduate research assistants from the electrical engineering and computer science department at UT-Knoxville who are participating in BR's projects, and both bring expertise from computer science areas to this research.

## Declarations

Publication of this article is supported by the National Institute for Computational Sciences, XSEDE funded by NSF and also supported in part by NSF grants EPS-0919436 and OCI-1053575.

This article has been published as part of *BMC Bioinformatics *Volume 14 Supplement 9, 2013: Selected articles from the 8th International Symposium on Bioinformatics Research and Applications (ISBRA'12). The full contents of the supplement are available online at http://www.biomedcentral.com/bmcbioinformatics/supplements/14/S9.
